# Targeted Nanocomplex Delivery for Protecting Vascular Integrity and Enhancing Anticancer Effects in Hepatocellular Carcinoma

**DOI:** 10.1002/smsc.202400616

**Published:** 2025-07-25

**Authors:** Hyelim Kim, Han Sol Lee, June Hong Ahn, Tae‐Wan Kwon, So‐Yeol Yoo, Donghyuk Seo, Sang Kyum Kim, Hee Ho Park, Seung‐Woo Cho, Wonhwa Lee, Jae‐Young Lee, Hong Nam Kim

**Affiliations:** ^1^ Brain Science Institute Korea Institute of Science and Technology (KIST) Seoul 02792 Republic of Korea; ^2^ Department of Biotechnology Yonsei University Seoul 03722 Republic of Korea; ^3^ College of Pharmacy Chosun University Gwangju 61452 Republic of Korea; ^4^ Division of Pulmonology and Allergy Department of Internal Medicine College of Medicine Yeungnam University and Regional Center for Respiratory Diseases Yeungnam University Medical Center Daegu 42415 Republic of Korea; ^5^ College of Pharmacy Chungnam National University Daejeon 34134 Republic of Korea; ^6^ College of Pharmacy and Research Institute of Pharmaceutical Sciences Seoul National University Seoul 08826 Republic of Korea; ^7^ Department of Chemistry Sungkyunkwan University Suwon Gyeonggi‐do 16419 Republic of Korea; ^8^ Department of Biotechnology College of Life Sciences and Biotechnology Korea University 145 Seoul 02841 Republic of Korea; ^9^ School of Mechanical Engineering Yonsei University Seoul 03722 Republic of Korea; ^10^ Yonsei‐KIST Convergence Research Institute Yonsei University Seoul 03722 Republic of Korea; ^11^ Division of Bio‐Medical Science & Technology KIST School University of Science and Technology Seoul 02792 Republic of Korea

**Keywords:** blood vessel protection, hepatocellular carcinoma, organ‐on‐a‐chip, targeted drug delivery

## Abstract

In cancer treatment, it is critical to enhance drug delivery efficiency to the target area while minimizing the side effects of chemotherapeutic drugs. Although intravenous administration of anticancer agents is commonly used, it often results in blood vessel damage and systemic side effects as well as poor targeted delivery. Therefore, developing new drug delivery formulations is essential to improve anticancer efficacy while reducing side effects. Herein, a nanohybrid methodology is presented to improve the targeted delivery of doxorubicin (DOX) for treating hepatocellular carcinoma (HCC) and to minimize vascular damage. An albumin–glucosamine (AG) lipid complex (LC), composed of stearyl glycyrrhetinate (SG) and DSPE‐PEG, is designed to serve as a liver cancer‐specific delivery system. The nanohybrid formulation, SGLC/DOX@AG, demonstrates significant anticancer activity and reduced side effects, as demonstrated by in vitro, in vivo, and cancer‐on‐a‐chip models. This study presents a novel carrier design and application model for drug formulation development and efficacy validation, providing insights into therapeutic development for HCC.

## Graphical abstract

Herein, a liver‐targeted nanohybrid formulation, SGLC/DOX@AG, is developed to enhance doxorubicin delivery and reduce vascular toxicity in hepatocellular carcinoma (HCC) treatment. Incorporating an albumin–glucosamine lipid complex, the system shows potent anticancer effects and minimized side effects across in vitro, in vivo, and cancer‐on‐a‐chip models. This platform offers a promising strategy for HCC‐specific drug delivery.

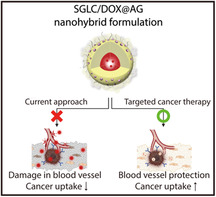

## 1 Introduction

Liver cancer is one of the most aggressive forms of cancer and remains a significant global health challenge. According to 2020 global cancer statistics, more than 9.0 million people were diagnosed with liver cancer with hepatocellular carcinoma (HCC) accounting for ≈90% of cases.^[^ ^1^ ^]^ The incidence rate is 3.6 times higher in males than females, and it is most prevalent among individuals in their 50s and 60s. The 5‐year survival rate of liver cancer, based on cases recorded between 2014 and 2018, is 37.8%, and this rate has been gradually increasing over time.^[^ ^2^ ^]^ Surgical resection is the primary treatment option for liver cancer; however, it carries the risk of postoperative complications, including hemorrhage, bile leakage, duct stenosis, reduced liver function, and liver failure.^[^ ^3^ ^]^ Radiation therapy is another effective treatment method, but it is often associated with various side effects, such as anorexia, vomiting, skin rash, gastrointestinal inflammation, and radiation pneumonitis.^[^ ^4^, ^5^ ^]^

The use of anticancer drugs is a straightforward approach to targeting liver cancer. However, since these drugs are delivered through the bloodstream, they circulate throughout the body, often causing unintended side effects, such as cardiotoxicity, severe tissue ulceration, and necrosis during chemotherapy.^[^ ^6^ ^]^ For example, the oral administration of sorafenib (an FDA‐approved drug in 2007 for treating advanced HCC with Child‐Pugh liver function class B; ClinicalTrials.gov identifier: NCT01405573) at a daily dose of 800 mg frequently leads to side effects like diarrhea and skin reactions.^[^ ^7^ ^]^ To minimize these adverse effects, various approaches have been developed. One approach involves using hollow chitosan–silica nanospheres for pH‐responsive drug delivery.^[^ ^8^ ^]^ Encapsulation of anticancer drugs helps reduce off‐target effects by limiting their accumulation in nontarget organs.^[^ ^9^ ^]^ Additionally, modification of surface moieties, such as sphingosine receptor‐targeted delivery, has been explored to improve specificity for liver cancer.^[^ ^10^ ^]^

However, these existing approaches focused primarily on delivery of anticancer drugs to solid tumors without considering the protection of blood vessels during administration. Damage to blood vessels is closely linked to the increased susceptibility to inflammation and infection in patients, with significantly reduced their survival rates during treatment.^[^ ^11^ ^]^ Protecting the integrity of blood vessels is essential to maintaining the patient's defense against inflammation and infection, as chronic inflammation can promote cancer cell progression including cell proliferation, invasion, and metastasis.^[^ ^12^, ^13^ ^]^ Furthermore, an inflammatory environment not only accelerates cancer progression but also heightens the risk of infection in surrounding tissues, thereby complicating disease management and negatively impacting patient outcomes.^[^ ^14^ ^]^

To enhance tumor‐targeting capability in liver cancer, we adopted a dual‐targeting strategy by incorporating albumin, glucosamine, and stearyl glycyrrhetinate (SG), all of which possess hepatotropism. Albumin, a natural plasma protein, accumulates in tumors via the enhanced permeability and retention effect and interacts with albumin‐binding proteins overexpressed in cancer cells.^[^ ^15^ ^]^ Glucosamine, an amino sugar, binds to glucose transporters (GLUTs) such as GLUT2, which are highly expressed in HCC cells, enabling selective uptake.^[^ ^16^ ^]^ In addition, SG, a lipophilic derivative of glycyrrhetinic acid, targets asialoglycoprotein receptors and glycyrrhizin receptors (GRs), both of which are abundantly expressed on hepatocytes and HCC cells.^[^ ^17^ ^]^ The combination of these ligands is expected to enhance active targeting and promote selective accumulation of drug carriers in liver tumors.

In this study, we present a cancer‐targeting drug carrier designed to efficiently deliver anticancer drugs to solid tumors, enhance anticancer effects, and protection of blood vessel structures during administration. To achieve this, we employed two key approaches: developing a drug carrier for the encapsulation of anticancer drugs and incorporation of liver cancer‐targeting moieties to enhance targeted delivery and endocytosis. The effectiveness of these approaches was evaluated using animal and vascularized liver cancer‐on‐a‐chip models. We focused on achieving targeted delivery of encapsulated anticancer drugs to liver, enhancing the efficacy of these drugs, protecting blood vessels, and reducing inflammation during administration. This integrated approach offers a novel strategy to improve the therapeutic efficacy of chemotherapy for liver cancer by improving targeted drug delivery, minimizing vascular damage, and reducing inflammation during treatment.

## 2 Results

### 2.1 Design of Nanohybrid Drug Carriers for Targeted Delivery to HCC

**Figure 1:**
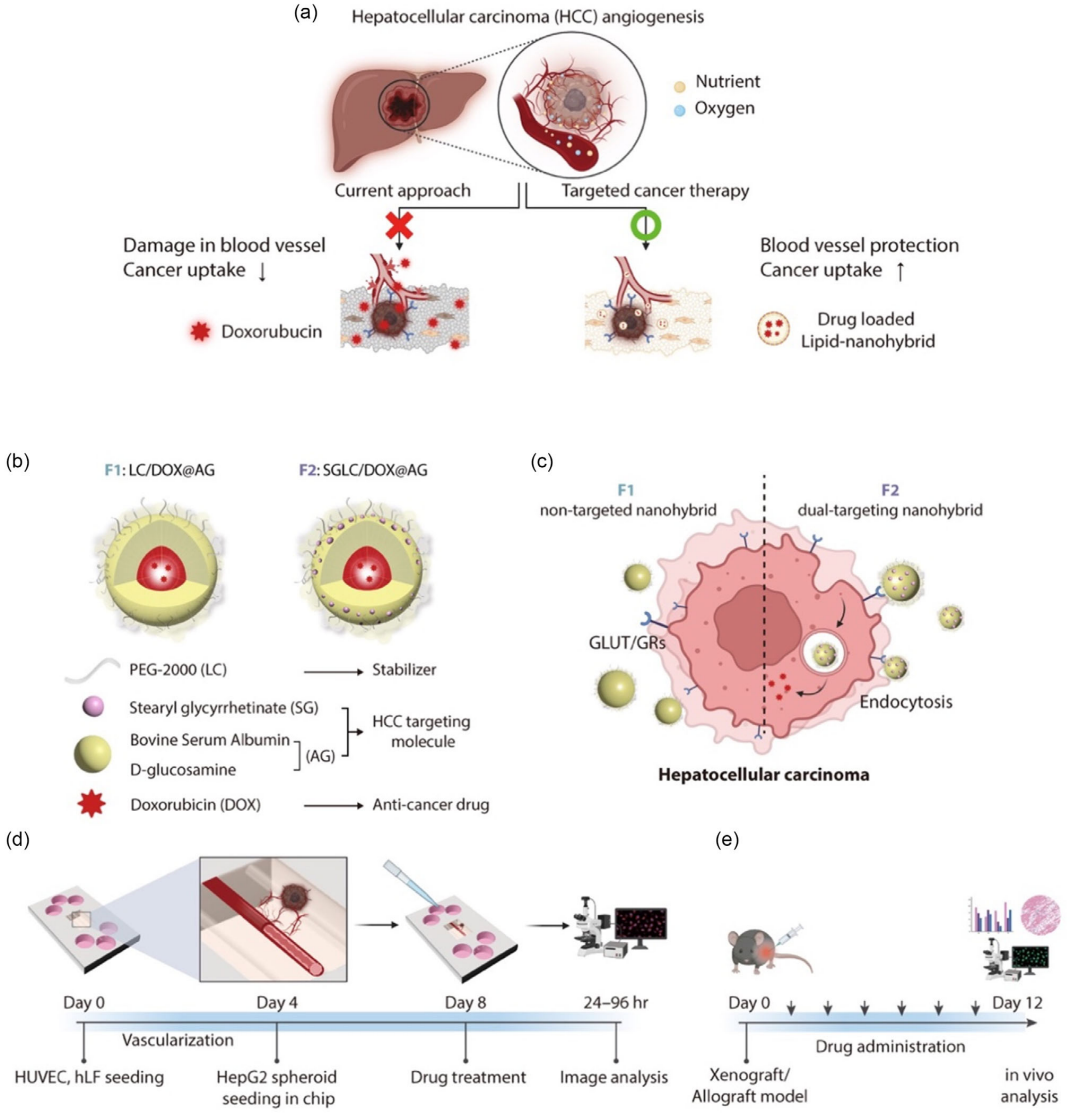
Design of dual‐targeting nanohybrids for targeted delivery to HCC. a) Schematic illustration of targeted drug delivery approach upon administration of the drug‐loaded carrier through the blood vessel. b) Schematic illustration of the developed nanohybrid with key materials for targeted delivery. F1 group has only liver‐targeting materials, while F2 group contains both liver‐targeting and cancer‐targeting moieties. c) Mechanism of nanohybrid uptake in HCC depending on the GLUT–GRs interactions. d) Timeline of experimental fabrication and analysis of vascularized cancer on a chip. e) HepG2 xenograft model analysis timeline. The drug (20 mg kg^−1^ DOX equivalent) was administered daily.

In this study, we focus on two primary objectives: 1) protecting normal blood vessels by reducing vascular–drug interactions and 2) enhancing anticancer effects through targeted drug delivery to solid tumors (**Figure** **1**a). To minimize the side effects of doxorubicin (DOX), we developed a nanohybrid drug carrier with a nanoparticulate morphology, comprising an albumin–lipid complex. This drug‐loaded nanohybrid system achieves dual entrapment of cytotoxic anticancer drugs by adsorbing DOX to albumin and further encapsulating it within a lipid complex, thereby minimizing unwanted exposure to nontarget tissues. Additionally, it features a cancer‐targeting moiety on its surface to enhance selective delivery. For specific targeting to the liver, we utilized albumin in the drug carrier, exploiting its natural affinity for liver tissues.^[^ ^18^ ^]^ Albumin's capacity to bind and transport hydrophobic nutrients, such as fatty acids, is also applicable to hydrophobic anticancer agents, thereby reducing direct cytotoxic drug exposure to blood vessels. Moreover, albumin has proven efficacy as a cancer drug delivery system, with lower immunogenicity and inherent tumor‐targeting capabilities, as demonstrated by ABRAXANE and FYARRO.^[^ ^19^, ^20^, ^21^ ^]^

To further enhance targeting, we incorporated glucosamine, a precursor of biochemical glycoprotein binding, into the drug carrier. Glucosamine, as a small molecule with high binding affinity for tumor cell surface receptors, increases uptake efficiency.^[^ ^22^, ^23^ ^]^ The combination of albumin and glucosamine facilitates targeted delivery to the GLUT receptors, which are upregulated in HCC due to increased glucose uptake and glycolysis.^[^ ^24^ ^]^ Additionally, the surface of the drug carrier was functionalized with SG, a ligand with affinity for GRs, which are overexpressed in HCC cells.^[^ ^25^ ^]^ SG is a stearyl ester form of 18 β‐glycyrrhetinic acid, a primary hydrolyzed composition of glycyrrhizic acid.^[^ ^26^, ^27^, ^28^ ^]^ Glycyrrhetinic acid has been used as a drug delivery material, including micelles, liposomes, and modified polymers (Figure 1b). Final formulation, which combines two liver‐targeting materials and an active tumor‐targeting moiety, is anticipated to enhance delivery specifically to HCC. The encapsulated cytotoxic drugs are expected to be selectively taken up by HCC cells through GLUT and GR pathways, releasing DOX intracellularly to inhibit tumor growth and minimize side effects (Figure 1c).

### 2.2 Validation of Targeted Delivery and Vascular Protection

To evaluate 1) the efficiency of targeted drug delivery and 2) the protection of blood vessels, we employed two experimental models: a vascularized cancer‐on‐a‐chip model and a liver cancer animal model (Figure 1d,e). The vascularized liver cancer‐on‐a‐chip model was developed by inducing sprouting angiogenesis in a three‐dimensionally cultured microvasculature.^[^ ^29^ ^]^ This model was constructed using human umbilical vein endothelial cells (HUVECs) and surrounded by human lung fibroblasts (HLFs), and the microvasculature was templated with microneedles. Directional angiogenesis was driven by interstitial flow, controlled by reservoir height differences. This setup enabled the formation of tumor‐associated capillaries sprouting from the primary blood vasculature, closely replicating in vivo tumor vasculature characteristics. This allowed detailed examination of drug carrier interactions with vascular structures.

For further assessment, we established a vascularized liver cancer model on an organ‐on‐a‐chip platform. HCC spheroids, precultured in 3D spheroid films for 4 days, were embedded in hydrogel and introduced into the chip through preformed microchannels (Figure 1d). Unidirectional interstitial flow, achieved by adjusting reservoir heights, facilitated tumor vascularization. Drug carriers were administered through the vascular network once sufficient tumor vascularization was confirmed (day 8). Confocal imaging verified the integration of capillary‐like blood networks with HCC spheroids, enabling precise evaluation of drug delivery and vascular protection. Additionally, two in vivo models were created: a xenograft mouse model was established by subcutaneous injection of HepG2 cells and maintained for 14 days, while an orthotopic mouse model was established by directly injecting mouse liver cancer cells into liver tissue. In both models, nanohybrids were administered intravenously every 3 days, with biodistribution across organs and tumor sizes monitored (Figure 1e). To closely mimic the drug administration process in humans, the drug‐loaded nanohybrids were delivered through blood vessels—via tail vein injection in the xenograft model and through reservoirs connected to the microvasculature in the cancer‐on‐a‐chip model. This approach allowed for a physiologically relevant evaluation of drug efficacy and side effects, effectively simulating human intravenous administration.

### 2.3 Preparation and Characterization of DOX‐Loaded Nanohybrids

In this study, we developed a series of DOX‐loaded nanohybrids to enhance targeted delivery to HCC. The albumin–glucosamine conjugate (AG) loaded with DOX, referred to as DOX@AG, served as the foundational structure. When DOX@AG was combined with the HCC‐targeting lipid complex (SGLC), it was designated as SGLC/DOX@AG (F2). For comparison, a formulation without the targeting moiety, LC/DOX@AG (F1), was prepared by complexing DOX@AG with lipids lacking SG. F1 is specific to liver tissues or liver tissue‐derived cells, whereas F2 targets both liver tissues and HCC cells.

**Figure 2:**
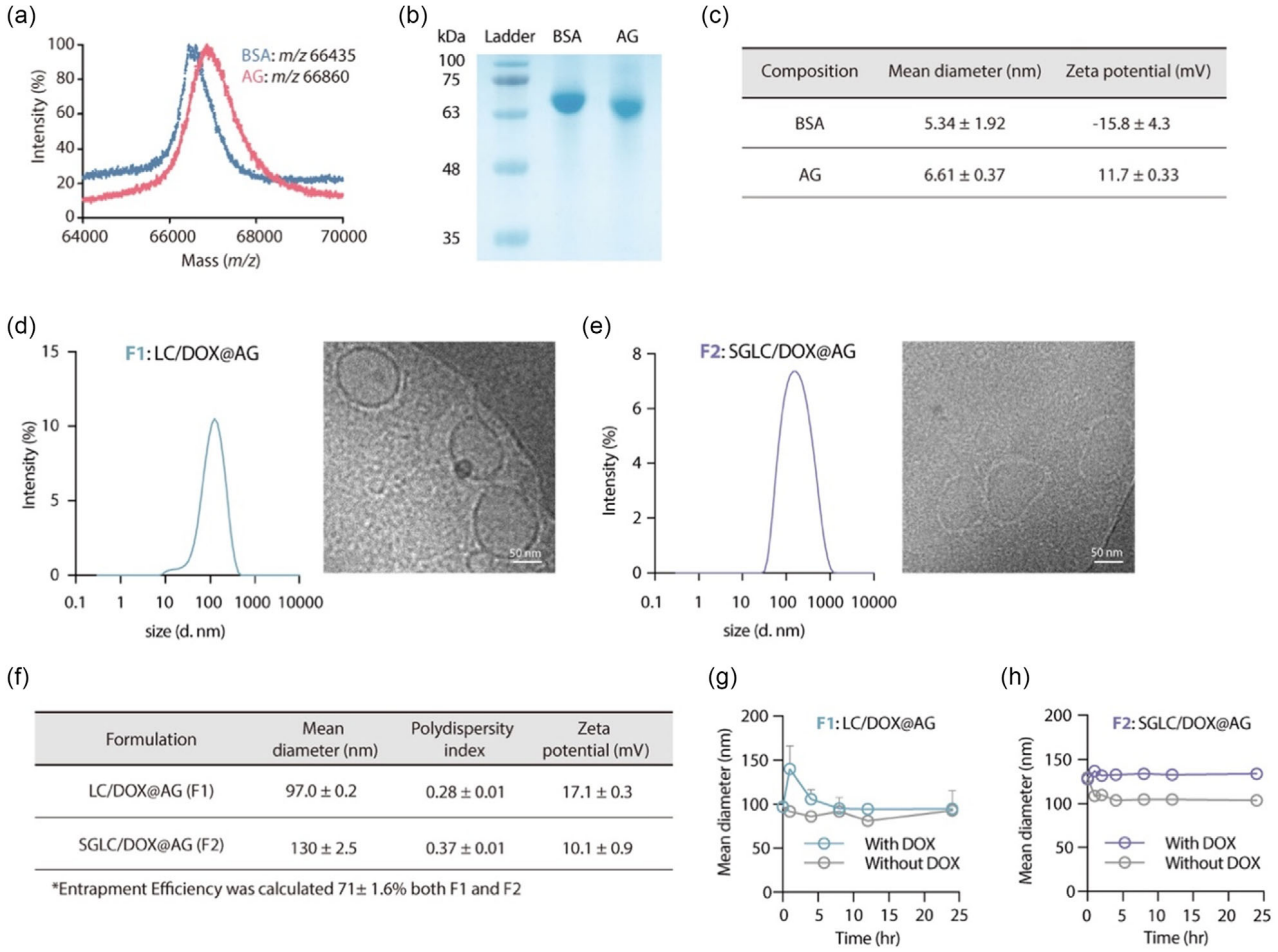
Properties of DOX‐loaded nanohybrids. a) MALDI–TOF analysis of BSA and AG for mass analysis. b) SDS–PAGE analysis of BSA and AG. c) Mean diameter and zeta potential of BSA and AG. d,e) Size distribution and TEM images of F1 (d) and F2 (e). Scale bar: 200 nm. f) Physical properties of F1 and F2, including mean diameter, polydispersity index, and zeta potential. g,h) Size stability of F1 (g) and F2 (h) in PBS (pH 7.4 and 37 °C) for 24 h. The diameter of F1 and F2 was maintained stably in pH 7.4 condition. All data are shown as mean ± SD (*n* = 3).

The synthesis of AG was achieved through an EDC/HOBt‐mediated reaction between bovine serum albumin (BSA) and d‐glucosamine, confirmed by matrix‐assisted laser desorption ionization–time of flight (MALDI–TOF) mass spectrometry. The resulting AG showed a mass‐to‐charge ratio (*m*/*z*) of 66 860, which is higher than that of BSA (*m*/*z* 66 435), indicating successful conjugation (**Figure** **2**a). This analysis estimated a molar conjugation ratio of glucosamine to albumin of ≈2.48. Sodium dodecyl sulfate–polyacrylamide gel electrophoresis (SDS–PAGE) analysis further corroborated this finding, as the AG band was broader than the BSA band (Figure 2b). The increase in zeta potential from −15.8 ± 4.3 mV for BSA to 11.7 ± 0.33 mV for AG supported the successful covalent attachment of glucosamine to albumin (Figure 2c). To load DOX, a hydrophobic anticancer agent, onto AG, we utilized a ball milling technique. This novel protein binding method facilitates efficient drug loading and induces self‐assembly of drug‐adsorbed proteins into stable nanoparticles in aqueous environments.^[^ ^30^ ^]^ The resulting DOX@AG complex served as the precursor for subsequent hybridization with lipids to create the final formulations. The encapsulation efficiency of DOX@AG was determined to be 74.0 ± 1.2%.

The DOX@AG formulation leverages the strong binding affinity of d‐glucosamine for GLUT receptors, which are highly expressed in liver tissue cells, thereby enhancing its targeting efficiency for liver tissues. Furthermore, albumin is known to bind to the 60 kDa glycoprotein (gp60), which is abundantly expressed on endothelial cells around tumor tissues, facilitating effective transport through receptor‐mediated transcytosis.^[^ ^31^, ^32^ ^]^ These characteristics are expected to significantly improve HCC targeting efficiency.

Although DOX@AG possesses notable advantages, we sought to further enhance its tumor targetability by focusing on GRs present in HCC. To this end, we employed a lipid complex comprising SG, egg phosphatidylcholine (EPC), and DSPE‐PEG‐NH_2_, which was then hybridized with DOX@AG, utilizing the inherent acyl chain‐binding property of albumin. SG was incorporated as a targeting moiety, capitalizing on its pivotal role in HCC targeting as previously mentioned.^[^ ^33^, ^34^, ^35^ ^]^ EPC was selected because of its prevalent use in drug delivery systems, both for its liver‐homing properties (i.e., through the recruitment of ApoE) and its capacity to enhance tumor penetration. Finally, DSPE‐PEG‐NH_2_ was added to ensure the formation of a nanocomplex with narrow size distribution and augmented stability within the bloodstream.

The lipid complex was successfully hybridized with DOX@AG, resulting in the formulation SGLC/DOX@AG (F2). For comparison, a control formulation, LC/DOX@AG (F1), was prepared using the same lipid complex, but without incorporating SG (Table S1, Supporting Information). Transmission electron microscopy (TEM) imaging revealed nanoscale particulate structures in both F1 and F2 (Figure 2d,e). Both F1 and F2 exhibited average hydrodynamic diameters of <130 nm with a narrow size distribution (polydispersity indices <0.37) and positive zeta potential, as observed by dynamic light scattering (DLS) analyses, suggesting that the nanohybrids may be suitable for intravenous administration (Figure 2f). A similar trend was observed in stability tests performed under high ionic strength conditions. Both F1 and F2 maintained their initial particle sizes for up to 24 h, and F1 and F2 without DOX also showed stable particle sizes (Figure 2g,h). Given their particle size and colloidal stability, the developed formulations validated their potential as nanoscale tumor‐targeted drug delivery platforms.^[^ ^36^ ^]^

### 2.4 In Vitro Release Test of LC/DOX@AG (F1) and SGLC/DOX@AG (F2)

**Figure 3:**
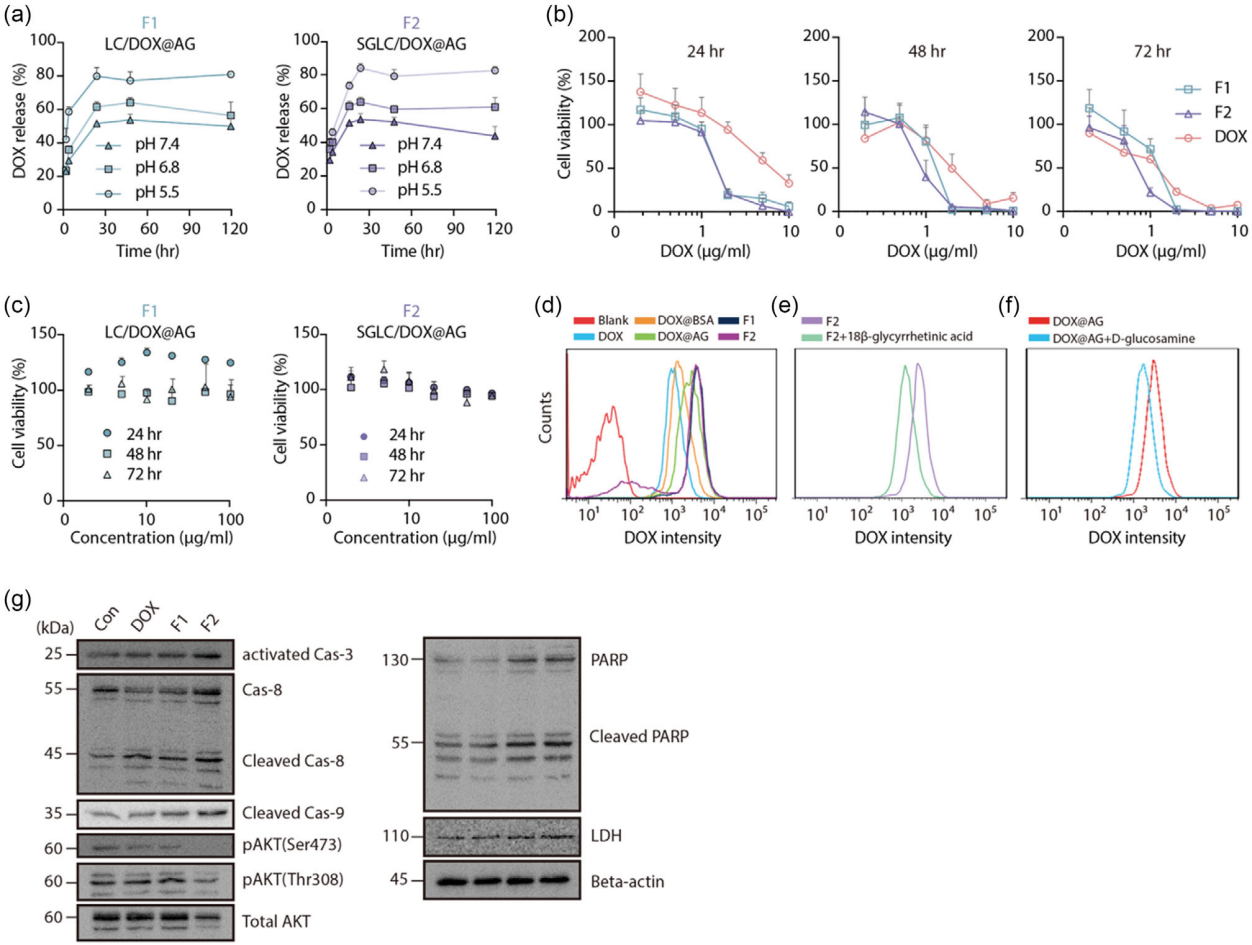
Validation of drug release and antitumor effects in vitro. a) In vitro DOX release profiles of F1 and F2 in PBS with pH 5.5, 6.8, and 7.4 for 96 h. b) In vitro cytotoxicity of DOX, F1, and F2 for 24, 48, and 72 h in 2D cultured HepG2 cells. IC_50_ of DOX, F1, and F2 were 6.66 ± 1.38, 1.54 ± 0.04, and 1.51 ± 0.12 μg mL^−1^, respectively. All data are shown as mean ± SD (*n* = 6). c) In vitro cytotoxicity of blank (drug unloaded) formation F1 and F2 in 2D cultured HepG2 cells. Cell viability (%) was calculated by CCK‐8‐based assay at specific nanohybrid concentrations (2, 5, 10, 20, 50, 100, and 200 μg mL^−1^) after incubation for 24, 48, and 72 h. All data are shown as mean ± SD (*n* = 6). d) Flow cytometry‐based analysis of intracellular uptake profiles. HepG2 cells treated with DOX and prepared DOX‐loaded nanohybrids were analyzed by flow cytometry after 4 h of incubation. e) Intracellular uptake profile of F2 and F2 with SG co‐treatment (75 μg mL^−1^). All data are shown as mean ± SD (*n* = 3), (*p* < 0.05). f) Intracellular uptake profile of DOX@AG and DOX@AG with d‐glucosamine co‐treatment (75 μg mL^−1^). g) Expression of apoptotic markers (Caspase‐3,8,9 and PARP) and cell proliferation markers (phosphorylated AKT [p‐AKT] and total AKT). All data are shown as mean ± SD (*n* = 3), (*p* < 0.05).

DOX release profiles of F1 and F2 were evaluated by a dialysis diffusion method in phosphate‐buffered saline (PBS) (pH 5.5, 6.8, and 7.4) at 37 °C, 50 rpm. DOX was gradually released over 24 h without a significant initial burst release, indicating a controlled release. Considering that nanoscale formulations usually accumulate in target tissues within 24 h, our nanohybrids seemed to have an appropriate release profile. The cumulative release of F1 and F2 reached ≈80% of the total drug, which could be attributed to the increased solubility of DOX at acidic pH, thus reducing hydrophobic interactions between the drugs and AG. Under more acidic conditions (pH 5.5), DOX was released more rapidly than physiological pH (7.4), which can be explained by the increased DOX solubility under acidic conditions. As the pH values of 5.5, 6.8, and 7.4 represent the endolysosomes, tumor microenvironment, and blood compartment (or normal tissues), respectively, the higher drug release under acidic conditions may enhance DOX exposure in tumor tissues compared to that in normal tissues (**Figure** **3**a).

### 2.5 DOX Release Profile and Cytotoxicity of Nanohybrids

In vitro cytotoxicity was evaluated by cell counting kit‐8 (CCK‐8)‐based proliferation assay in 2D‐cultured HepG2 cells. Since F2 can be taken up by more receptors (i.e., GLUTs and GRs), F2 showed significantly higher cytotoxicity than the DOX solution and F1 (Figure 3b). IC50 values of DOX, F1, and F2 on HepG2 cells after 24 h of incubation were 6.66 ± 1.38, 1.54 ± 0.04, and 1.51 ± 0.12 μg mL^−1^, respectively. According to the 24 h results, both F1 and F2 exhibited higher anticancer efficacy than the DOX solution (*p* < 0.05).

Despite the different cellular uptake mechanisms of the DOX solution and DOX‐loaded nanohybrids (passive diffusion vs receptor‐mediated endocytosis), the developed nanohybrid displayed superior DOX cytotoxicity. Notably, a significantly higher anticancer efficacy of F2 than F1 was observed after 48 h (1.17 ± 0.07 μg mL^−1^ [F1] vs 0.94 ± 0.15 μg mL^−1^ [F2]) or 72 h (1.12 ± 0.08 μg mL^−1^ [F1] vs 0.70 ± 0.10 μg mL^−1^ [F2]) incubation (*p* < 0.05) (Table S2, Supporting Information). To confirm whether the formulation was toxic, cell viability was observed for the two formulations without DOX. Under these conditions, blank formulations (without DOX loading) did not show any notable cytotoxicity (Figure 3c). In particular, the negligible cytotoxicity of the blank formulations guaranteed their successful application as safe, tumor‐targeted drug delivery systems. Consistent with these findings, additional data from multiple HCC cell lines (HepG2, Hep3B, and Huh7) confirm that F2 significantly reduces cell viability more effectively than F1 and free DOX across a range of concentrations (Figure S1a, Supporting Information). The enhanced cytotoxicity of F2 is attributed to its dual‐targeting mechanism, which facilitates higher cellular uptake in HCC cells. Confocal microscopy revealed enhanced nuclear accumulation of DOX with F1 and F2 formulations compared to free DOX and controls (Figure S1b, Supporting Information).

### 2.6 Enhanced Cellular Uptake and Targeting Efficiency of F2

In vitro cellular uptake of DOX was observed using flow cytometry after 4 h of incubation with the DOX solution and various formulations. As expected, F2 exhibited a significantly higher cellular uptake, as indicated by DOX fluorescence intensity, than the other formulations (Figure 3d). This result correlated with the higher cytotoxicity of F2 in 2D cultured cancer cells, suggesting that the cancer‐targeting ligand in F2 for cancer‐displaying receptors (GLUTs and GRs) plays an important role in cellular uptake.

Importantly, coadministration of free SG (75 μg mL^−1^) with F2 reduced the cellular uptake of F2 to 62.1 ± 0.55% compared to F2‐only case (Figure 3e). This result supports the idea that SG decorated on nanohybrid function well as a targeting moiety. In addition, cotreatment of d‐glucosamine (competitive inhibitor; 75 μg mL^−1^) with DOX@AG showed lower fluorescence intensity than DOX@AG (Figure 3f). This result indicated that AG promoted the binding of nanohybrids to GLUTs. In these assays, concentrations of SG and d‐glucosamine were selected within the nontoxic range.^[^ ^37^, ^38^ ^]^ Collectively, these results indicated that d‐glucosamine and SG successfully acted as HCC‐targeting moieties.

### 2.7 Mechanistic Insights into Apoptotic Pathways Induced by Nanohybrids

To further elucidate the mechanisms underlying the enhanced antitumor effects of the nanohybrids, western blot analyses were conducted to assess apoptosis markers. Both F1 and F2 induced apoptosis, significantly enhanced the cleavage of key apoptotic regulators, including caspase‐3, caspase‐8, and caspase‐9. These observations strongly emphasize the activation of apoptosis, a fundamental process leading to programmed cell death.^[^ ^39^ ^]^ Concurrently, administration of F1 and F2 induced a notable reduction in AKT phosphorylation at both Ser473 and Thr308 (Figure 3g, left panel), highlighting the inhibition of the prosurvival AKT pathway,^[^ ^40^, ^41^ ^]^ further promoting the apoptotic response. Additionally, our study revealed a substantial increase in both the expression and cleavage of poly(ADP‐ribose) polymerase (PARP), a protein involved in DNA repair and apoptosis.^[^ ^42^ ^]^ Furthermore, F1 and F2 treatment significantly increased lactate dehydrogenase (LDH) levels, a well‐established biomarker of cellular damage and apoptosis. (Figure 3g, right panel).^[^ ^43^ ^]^ Collectively, these findings demonstrate that F2, with its dual‐targeting design, provides superior delivery, uptake, and therapeutic efficacy against HCC, highlighting its potential as a promising candidate for targeted liver cancer therapy.

### 2.8 3D Spheroid Model for Drug Penetration and Therapeutic Efficacy

**Figure 4:**
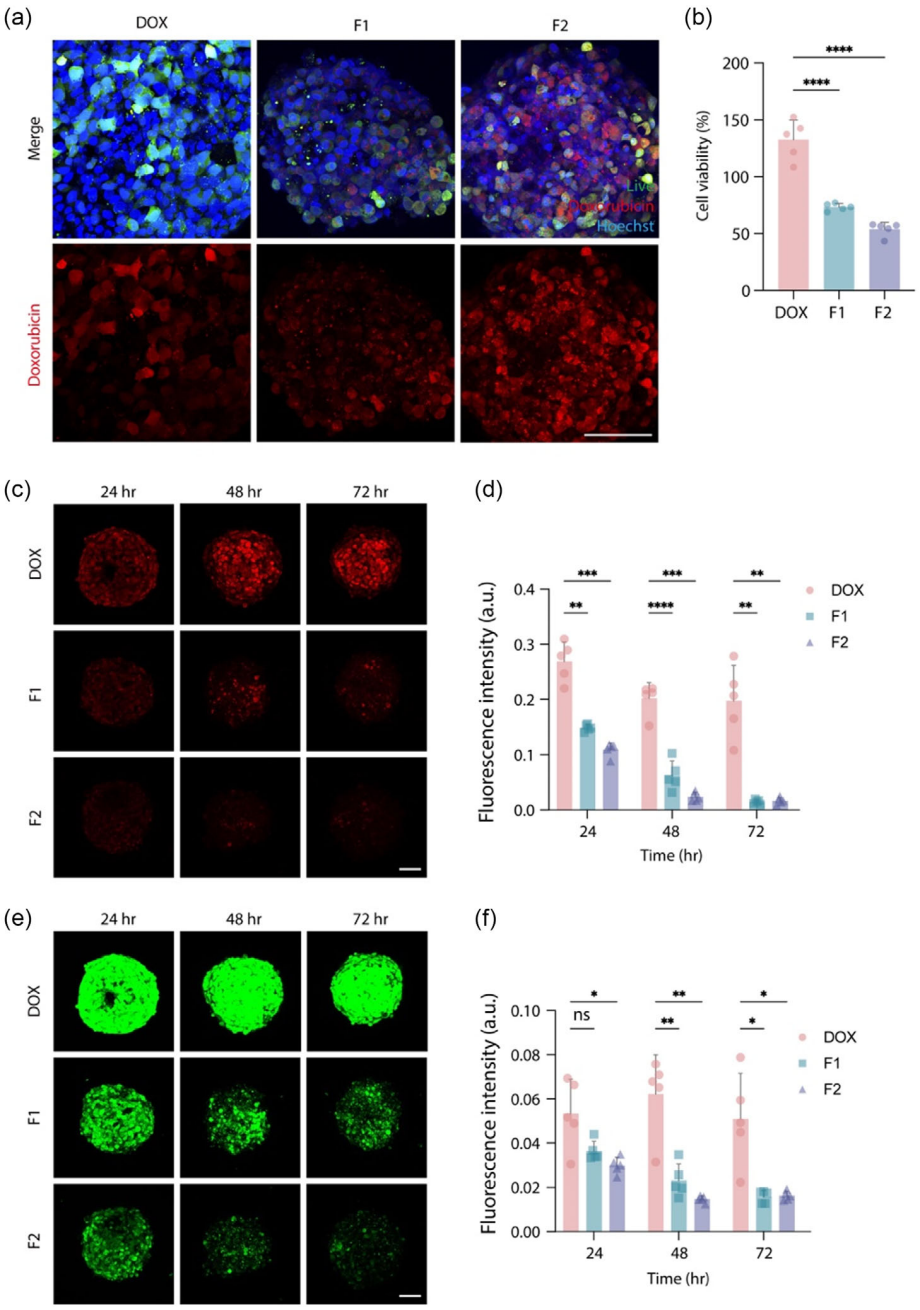
Antitumor effect of DOX, F1 and F2 in HepG2 spheroid a) Confocal images of DOX uptake in HepG2 spheroid at 24 h. b) Relative cell viability of HepG2 spheroid after 24 h treatment with DOX, F1 and F2. c) Confocal images of DOX uptake at 24, 48 and 72 h. d) Fluorescence intensity of DOX levels in HepG2 spheroid at 24, 48 and 72 h. e) Confocal images of Calcein‐AM staining at 24, 48 and 72 h. f) Fluorescence intensity of Calcein‐AM levels in HepG2 spheroid at 24, 48 and 72 h. All data are presented as mean ± standard error of the mean (SEM). *P*‐values are calculated using an ANOVA. *p < 0.05, **p < 0.01. ***p < 0.01. ****p < 0.01. Scale bar, 100 μm.

To assess drug penetration and therapeutic efficacy in 3D settings, HepG2 spheroids were used to mimic physiologically relevant conditions, and cell viability was evaluated. Confocal microscopy revealed that drug accumulation (DOX, red) in cells was more effective in the F1 and F2 groups compared to the DOX group (**Figure** **4**a). Interestingly, DOX delivered via nanohybrids (F1 and F2) exhibited a punctate and vesicular intracellular distribution pattern, suggesting endocytosis‐mediated uptake, in contrast to the diffuse nuclear localization observed with free DOX.^[^ ^44^ ^]^

Cell viability was measured by staining with Calcein‐AM (green). A comparison with the DOX group revealed that both the F1 and F2 groups exhibited a decrease in cell viability, with the F2 group demonstrating a markedly enhanced anticancer effect (Figure 4b). To evaluate drug accumulation, we measured DOX (red) fluorescence intensity at 24, 48, and 72 h using confocal microscopy (Figure 4c). Quantitative analysis of fluorescence intensity revealed that the F1 and F2 groups exhibited reduced fluorescence compared to the DOX group (Figure 4d).

Calcein‐AM (green) staining of live cells showed a decrease in fluorescence intensity in the F1 and F2 groups at all time points (24, 48, and 72 h) (Figure 4e). Notably, the F2 group demonstrated the lowest fluorescence intensity across all time points, indicating that cell death was induced after 24 h (Figure 4f). Despite the lower DOX signal (red) observed in the F1 and F2 groups (Figure 4c,d), these groups achieved more effective cancer eradication with smaller amount of DOX exposure. This suggests a highly efficient anticancer effect driven by targeted drug delivery.

### 2.9 Evaluation of Nanohybrid Efficacy Using Vascularized Cancer‐on‐a‐Chip Model

To assess the efficacy of the nanohybrids (F1 and F2) in a controlled microenvironment, a vascularized cancer‐on‐a‐chip model was utilized to replicate the complex interactions between tumor cells and the surrounding vasculature. HUVECs and HLFs were seeded to induce angiogenesis, followed by HepG2 spheroid seeding on day 4. Treatments with DOX, F1, and F2 were administered on day 8, with subsequent analysis conducted after 24 h (**Figure** **5**a).

Figure 5Evaluation of vascular disruption and inflammation in vascularized cancer on a chip. a) Timeline of vascularized cancer on a chip model. b) Vascularized blood vessels and HCC spheroids within a cancer‐on‐a‐chip model. c) HCC spheroid growth curve in angiogenesis chip model. F2 showing the least growth during the treatment period. d) Image of transmitted 40 kDa FITC‐dextran through the angiogenesis blood vessels after DOX, F1, F2 treatment for 24 h. e) Quantified transendothelial permeability of 40 kDa FITC‐dextran from the microvasculature in F1‐ and F2‐treated groups was lower than that in the DOX‐treated group. f) Immunofluorescence imaging of vascular inflammation marker (ICAM‐1). g) Quantification of ICAM‐1 fluorescence intensity. (*n* ≥ 4 for each group) h) Expression of blood vessel junction marker (VE‐cadherin). i) Quantitative fluorescence intensity of VE‐cadherin. (*n* = 3 for each group) (significance is indicated by *** for *p *< 0.005; all by unpaired *t*‐test). The experiment was performed at least three times with replicates. Data are presented as mean ± standard error of the mean (SEM). *P*‐values are calculated using an ANOVA (A–H). *p < 0.05, **p < 0.01. ***p < 0.01. ****p < 0.01. Scale bar, 100 μm.

**Figure:**
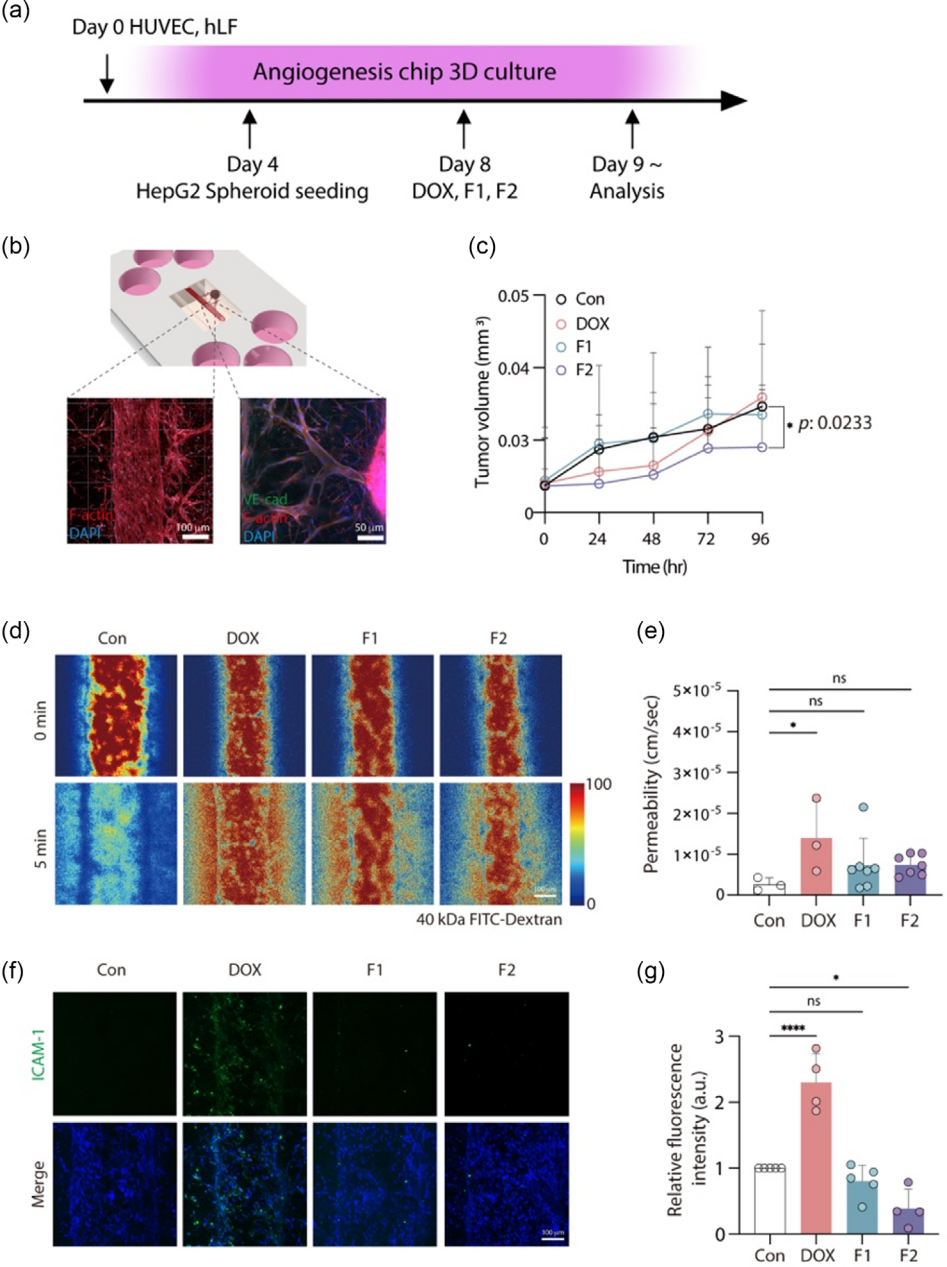

**Figure:**
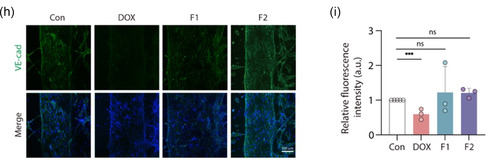

To confirm the successful formation and sprouting of blood vessels into the tumor spheroids, immunofluorescence staining for F‐actin and VE‐cadherin was performed. Confocal images demonstrated that the endothelial cells proliferated effectively, forming tube formation, sprouted toward the tumor spheroids and formed microvessels, as indicated by the distinct staining patterns for F‐actin (red) and VE‐cadherin (green) (Figure 5b). This setup allowed for the evaluation of drug interactions with both tumor cells and the surrounding vascular network in a physiologically relevant environment. Tumor volume measurements indicated that both F1 and F2 effectively inhibited tumor growth compared to the control (Con) and free DOX treatments, with F2 showing a statistically significant reduction in tumor volume (Figure 5c). This finding underscores the enhanced therapeutic efficacy of F2, likely due to its dual‐targeting capability.

### 2.10 Evaluation of Protection of Blood Vessels Using Vascularized Cancer‐on‐a‐Chip Model

To evaluate the impact of the nanohybrids on vascular integrity, permeability assays were conducted using 40 kDa FITC‐dextran. Confocal microscopy images showed that treatment with free DOX resulted in significantly increased vascular permeability, indicating potential damage to the endothelial barrier. In contrast, treatments with F1 and F2 preserved vascular integrity, as evidenced by lower permeability values (Figure 5d). Quantitative analysis confirmed that both F1 and F2 significantly reduced vascular permeability compared to DOX, with F2 demonstrating the most substantial protective effect (Figure 5e). Further analysis in the absence of HCC cells showed similar trends in preserving vascular integrity. Permeability assays conducted in the absence of HCC revealed that free DOX still increased permeability, whereas F1 and F2 treatments maintained lower permeability levels, similar to the control (Figure S2a,b, Supporting Information). This suggests that the protective effect of F2 on the vasculature is not solely dependent on the presence of tumor cells but also effective in maintaining endothelial barrier function under normal conditions. Overall, these results suggest that F1 and F2, particularly F2, can protect blood vessels from DOX‐induced damage.

Vascular inflammation was assessed through immunofluorescence staining for intercellular adhesion molecule‐1 (ICAM‐1). The DOX‐treated group exhibited a marked increase in ICAM‐1 expression, indicating a strong inflammatory response. In contrast, F1 and F2 treatments resulted in significantly lower ICAM‐1 levels, comparable to the control group (Figure 5f). Quantification of fluorescence intensity confirmed that F1 and F2 significantly reduced inflammation, with F2 showing the lowest ICAM‐1 expression levels (Figure 4g). This indicates that F2 effectively mitigates DOX‐induced vascular inflammation. Similarly, in conditions without HCC, ICAM‐1 expression was significantly elevated in the DOX‐treated group, while F1 and F2 maintained low levels of ICAM‐1, similar to the control (Figure S2c,d, Supporting Information). These findings reinforce the anti‐inflammatory properties of F2 and its capacity to protect the vasculature, even in the absence of tumor‐induced stress.

Endothelial junction functionality was further evaluated through immunostaining for VE‐cadherin, a critical component of vascular integrity. The DOX‐treated group showed a marked reduction in VE‐cadherin expression, indicating compromised endothelial junctions. Conversely, F1 and F2 treatments preserved VE‐cadherin expression, suggesting maintained endothelial integrity (Figure 5h). Quantitative analysis supported these observations, with F2 demonstrating significantly better preservation of endothelial junctions compared to the DOX‐treated group (Figure 5i). Overall, these results demonstrate that the dual‐targeting nanohybrid (F2) not only enhances antitumor efficacy but also provides protection to vascular structures, reducing chemotherapy‐induced damage, inflammation, and preserving endothelial integrity.

### 2.11 In Vivo Efficacy of Nanohybrids in Xenograft and Orthotopic Liver Cancer Mouse Models

The in vivo efficacy of LC/DOX@AG (F1) and SGLC/DOX@AG (F2) nanohybrids was evaluated using xenograft and orthotopic liver cancer mouse models to assess their tumor‐targeting capabilities, therapeutic efficacy, and safety profiles. In the xenograft model, subcutaneous HepG2 tumors were established in mice, and the formulations were administered intravenously. For in vivo fluorescence imaging, Cy5.5‐conjugated AG was used to prepare the nanohybrids (F1 and F2), as described in the Experimental Section. In vivo fluorescence imaging demonstrated that F2 exhibited superior tumor localization and retention compared to F1 and free DOX, with higher fluorescence intensity sustained within the tumor region over time (**Figure** **6**a). Ex vivo imaging further confirmed that F2 showed preferential accumulation in tumors with minimal off‐target distribution in major organs such as the liver, kidneys, spleen, lungs, and heart (Figure 6b). Quantitative analysis of radiant efficiency indicated that F2 maintained significantly higher tumor retention levels up to 24 h postinjection compared to F1 and controls (Figure 6c).

Figure 6
In vivo antitumor efficacy and targeting ability of nanohybrids in xenograft and orthotopic HCC models. a) In vivo imaging of HepG2 xenograft‐bearing mice after intravenous injection of fluorescently labeled nanohybrids (F1: LC/DOX@AG; F2: SGLC/DOX@AG), with the AG group (Cy5.5‐labeled AG without lipids) serving as a control. Images were captured at 1, 2, 6, and 24 h postinjection to evaluate the targeting ability of the nanohybrids. b) Ex vivo biodistribution analysis of major organs and tumors excised from the xenograft model 24 h postinjection, highlighting the tumor‐specific accumulation of the nanohybrids. c) Quantitative analysis of radiance efficiency over 24 h, comparing the retention of nanohybrids in the tumor site. d) Representative images of excised tumors from each treatment group (Control, DOX, F1, and F2) after 12 days of treatment. e) Tumor growth curves showing the increase in tumor volume over time in each treatment group. f) Final tumor weights measured at the end of the treatment period, demonstrating the significant reduction in tumor burden in the F2‐treated group. g) Western blot analysis of key apoptotic and antiapoptotic markers (BCL‐2, Caspase‐9, Caspase‐1, and AKT) in tumor tissues from each treatment group, indicating the induction of apoptosis in F2‐treated tumors. h) Immunohistochemistry (IHC) staining for EpCAM (green), BrdU (red), and DAPI (blue) in tumor tissues from an orthotopic HCC model established using the Hepa1‐6 cell line. Merged images show the colocalization and reduced expression of these markers in the F2‐treated group. H&E staining of tumor tissues at 10× and 20× magnification shows extensive necrosis in the F2‐treated group. i,j) Quantification of EpCAM and BrdU fluorescent intensity, showing a significant reduction in the F2‐treated group compared to control, DOX, and F1 groups. k) Tumor size measurements in the orthotopic model at the end of the treatment period, highlighting the marked reduction in tumor size in the F2‐treated group. l) Kaplan–Meier survival curve indicating significantly improved survival rates in the F2‐treated group compared to other treatments (*p* = 0.0312).

**Figure:**
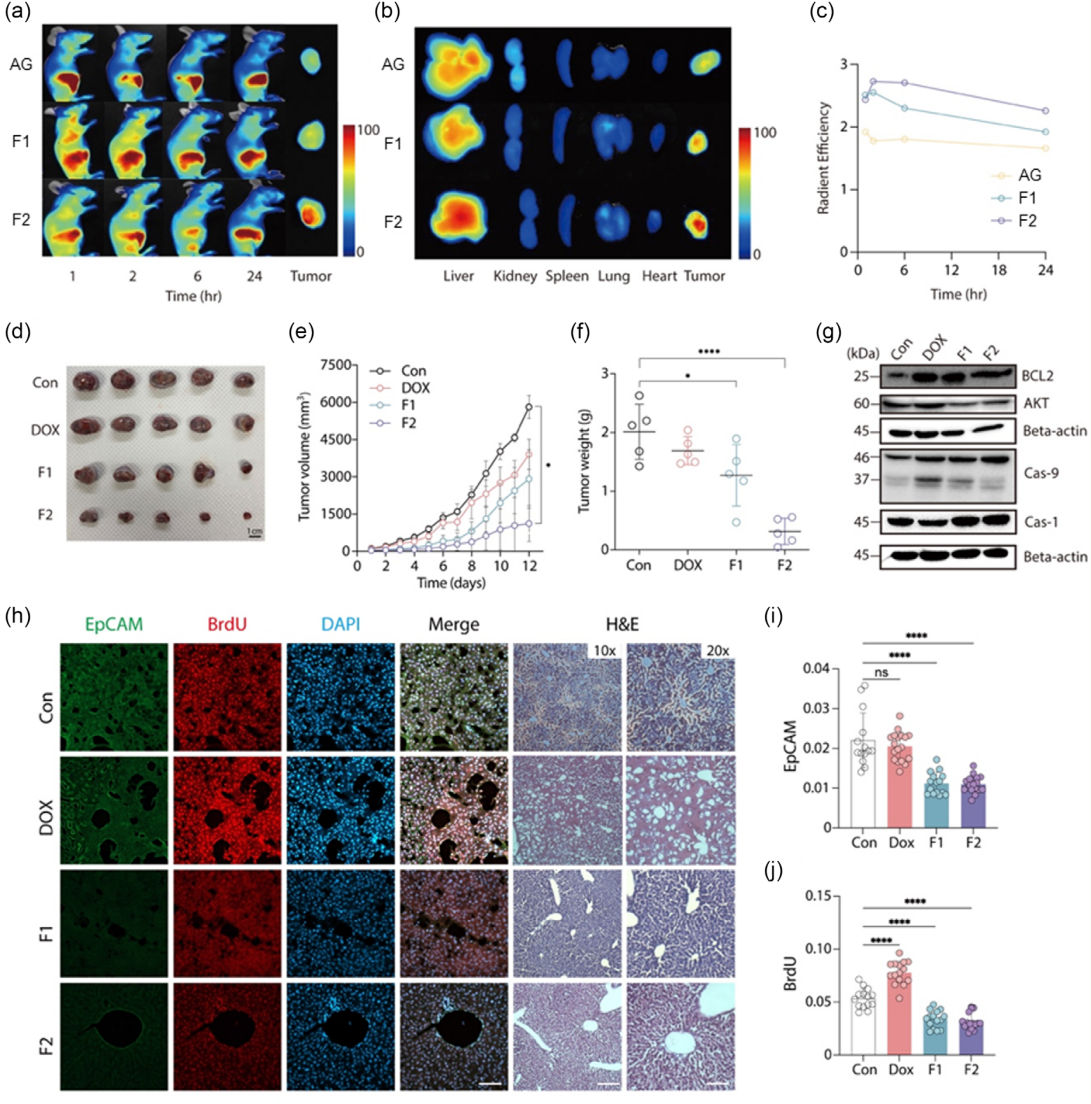

**Figure:**
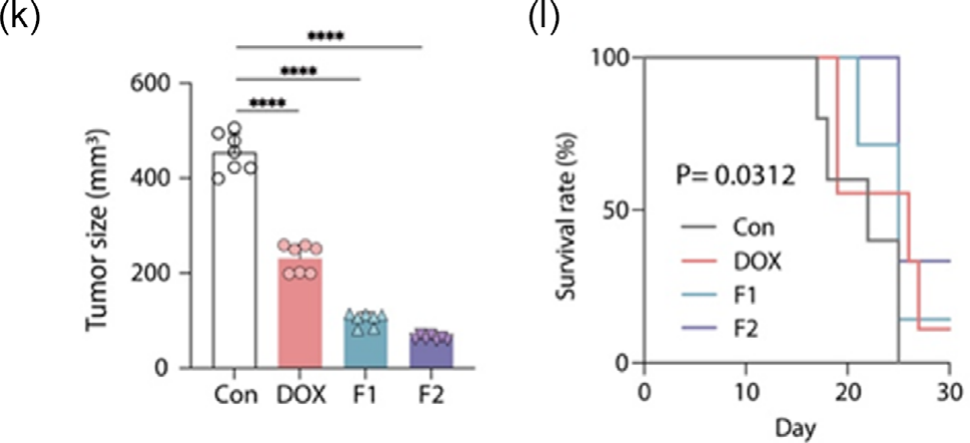

Tumor growth inhibition was assessed by measuring tumor volume and weight. Both F1 and F2 significantly reduced tumor growth compared to control and free DOX groups, with F2 showing the most pronounced reduction in tumor volume (Figure 6d,e). The excised tumors from the F2‐treated group were also significantly lighter in weight, confirming the enhanced therapeutic efficacy of F2 (Figure 6f). Additionally, body weight monitoring revealed that mice treated with F2 maintained stable body weights throughout the study period, indicating a lower systemic toxicity compared to the DOX‐treated group, which showed slight weight loss (Figure S3a, Supporting Information). To further evaluate systemic toxicity, blood samples were analyzed for markers of organ damage, such as alanine aminotransferase (ALT), aspartate aminotransferase (AST), blood urea nitrogen (BUN), and creatinine levels, which reflect liver and kidney function respectively (Table S3, Supporting Information). The results demonstrated that the levels of these markers remained within normal ranges for both F1 and F2 groups, suggesting minimal organ toxicity. In contrast, mice treated with free DOX exhibited significantly elevated levels of ALT and AST, indicating liver damage, as well as increased BUN and creatinine levels, suggesting impaired kidney function.

These findings align with the histopathological analysis using hematoxylin and eosin (H&E) staining, where the DOX‐treated group displayed significant tissue disruption and signs of necrosis, while the F1 and F2 groups showed more preserved tissue architecture. Notably, F2 demonstrates the least disruption, indicative of lower toxicity and reduced side effects (Figure S3b, Supporting Information). Molecular analysis revealed that F2 treatment led to a notable decrease in BCL‐2 and AKT expression, key markers associated with cell survival, and an increase in cleaved caspase‐9 and caspase‐3, indicating enhanced apoptosis induction compared to F1 and free DOX (Figure 5g). This comprehensive assessment underscores that F2, with its dual‐targeting strategy, not only achieves superior tumor specificity and enhanced antitumor efficacy but also minimizes systemic toxicity, making it a promising candidate for targeted liver cancer therapy.

In the orthotopic liver cancer model, where Hepa1‐6 cells were directly injected into the liver to better mimic the native tumor microenvironment, immunohistochemical analysis for EpCAM and BrdU showed significant reductions in these markers in the F2‐treated group, indicating decreased tumor cell proliferation and stemness (Figure 6h–j). Tumor size measurements confirmed that F2 treatment resulted in the smallest tumors among all groups (Figure 6k).

Furthermore, Kaplan–Meier survival analysis demonstrated that F2 significantly extended survival compared to other groups, underscoring its potential to improve therapeutic outcomes in liver cancer treatment (Figure 6l). These results collectively demonstrate that F2, with its dual‐targeting strategy, achieves superior tumor specificity, enhanced antitumor efficacy, and improved survival rates in both xenograft and orthotopic liver cancer models, highlighting its promise as a targeted liver cancer therapy.

## 3 Discussion

DOX is a widely used antitumor drug for the treatment of solid tumors. However, DOX has inherent side effects, including heart problems and damage to normal cells. Therefore, encapsulation and targeted delivery are essential for successful patient care. Accordingly, we have developed nanoparticulate formulations that combine DOX and albumin. Albumin‐based carriers circulate throughout the body and tend to accumulate in the liver. This approach, similar to clinical drugs like ABRAXANE, still faces challenges regarding liver cancer specificity. In recent studies, glucosamine has shown affinity for the surface receptors of tumor cells, and SG, a glycyrrhetinic acid derivative, has also been studied as an antitumor therapy.^[^ ^22^, ^23^ ^]^ In this study, d‐glucosamine and SG were used for the uptake of nanohybrid‐encapsulated drugs in liver cancer via GLUTs and GRs. Indeed, DOX‐loaded nanohybrids with SG decoration demonstrated targeted delivery, both in vivo and in vitro. Furthermore, the nanohybrid‐mediated delivery of DOX significantly reduces vascular disruption and inflammation.

We utilized a microphysiological model developed to mimic the physiology and pathology of human tissues. To recapitulate the drug delivery route and pathophysiology of the tumor microenvironment, we devised a vascularized tumor model. Microphysiological systems have drawn considerable attention as alternative models for drug screening.^[^ ^45^ ^]^ This model reflects drug delivery through blood vessels, allowing drug injections to be studied in a physiologically relevant manner. Because the sprouted capillaries made direct contact with the HCC spheroid, the nanohybrids were able to reach and bind to the GRs. The transported DOX‐loaded drug carriers are taken up via endocytosis, which consequently induces antitumor effects in tumor cells. Superior antitumor effects have been reproducibly observed in both in vivo and microphysiological models. However, while this platform successfully reproduces key aspects of perfusion and drug diffusion, it does not explicitly incorporate immune interactions, stromal remodeling, or dynamic matrix stiffness. These factors are known to substantially influence therapeutic responses and tumor progression. Future studies will focus on integrating immune cell populations and tunable extracellular matrices to improve the predictive relevance and comprehensiveness of the model.

The efficacy of our formulation and its reduced side effects were evaluated in a xenograft model. In this animal model, the F2 formulation showed targeted delivery to solid tumors, increased antitumor effects, and protection of blood vessels and normal tissues. These experimental results correlate well with those obtained from a vascularized tumor platform, suggesting the reliability of our drug delivery system for translation. Importantly, preserving vascular integrity during treatment is clinically significant in HCC. Vascular disruption can impair tumor perfusion, foster hypoxia, and trigger pro‐inflammatory signals that promote tumor progression and immune evasion. By maintaining endothelial barrier function, our nanohybrids may facilitate more uniform drug distribution, support immune infiltration, and ultimately improve therapeutic outcomes. We also evaluated the upregulation of apoptosis‐related markers in cancer cells from animal models, vascularized tumor chips, and 2D cultured cancer cells. Western blot analysis showed that tissues or cells exposed to the F2 formulation exhibited increased levels of apoptosis‐related markers, including increased caspase series, decreased pAKT, and increased PARP levels. Such enhanced antitumor effects originate from the increased uptake of DOX via targeted delivery. These results provide valuable insights into the development of advanced drug formulation strategies, emphasizing their potential to enhance therapeutic efficacy and targeting precision.

## 4 Conclusion

In this study, we demonstrated that DOX‐loaded nanohybrids enable targeted delivery to HCC, enhancing drug accumulation while reducing side effects. To this end, three methodologies have been adopted: in vitro, in vivo, and chip‐based models. Targeted delivery efficiency and reduced side effects were confirmed in a xenograft mouse model and a vascularized tumor model. In both models, our nanohybrids composed of liver‐targeting components and functionalized with tumor‐specific ligands demonstrated improved uptake efficiency and antitumor efficacy. Furthermore, the targeted delivery method suppressed undesirable side effects, such as vascular damage, and preserved endothelial barrier integrity, which is particularly important for maintaining perfusion and limiting proinflammatory signaling in liver cancer therapy. While additional work integrating immune interactions and dynamic stromal elements would further strengthen the translational relevance of the model, our approach provides a promising strategy to address current unmet needs in targeted antitumor drug delivery.

## 5 Experimental Section

5.1

5.1.1

##### Materials

Bovine serum albumin (BSA), 1‐ethyl‐3‐(3‐dimethyl aminopropyl) carbodiimide hydrochloride (EDC HCl), d‐glucosamine, and 1‐hydroxybenzotriazole hydrate (HOBt) were obtained from Sigma–Aldrich Co. (St. Louis, MO, USA). DOX‐free base was purchased from MedKoo Biosciences (Morrisville, NC, USA). SG was purchased from Tokyo Chemical Industry Co., LTD. (Tokyo, Japan). 1,2‐distearoyl‐*sn*‐glycero‐3‐phosphoethanolamine‐*N*‐[amino(polyethylene glycol)‐2000] was purchased from BioActs (Incheon, Republic of Korea). Lipoid E100 (egg phosphatidylcholine) was purchased from Lipoid GmbH (Ludwigshafen, Germany).

##### Synthesis of AG Conjugate

BSA (100 mg, 1.5 μmol) was dissolved in double‐deionized water (DDW; 150 mL), followed by the addition of EDC HCl (35 mg, 225 μmol), HOBt (31 mg, 225 μmol), and d‐glucosamine (66 mg, 300 μmol) with stirring. After being stirred for 24 h at 5 °C under light protection, the reaction mixture was dialyzed in a dialysis bag (molecular weight cutoff: 3500 Da; Cellu Sep, Membrane Filtration Products, Seguin, TX, USA) against DDW for 24 h. The dialysis product, AG conjugate, was lyophilized at –70 °C for further use.

##### Characterization of AG

The successful synthesis of AG was confirmed by MALDI–TOF mass spectrometry (Voyager DE‐STR, Applied Biosystems Inc., Waltham, MA, USA) and SDS–PAGE. Each sample (1 mg mL^−1^) was loaded onto a 12% SDS–PAGE gel. The zeta potential of AG was determined using a Zetasizer Ultra (Malvern Panalytical, Worcestershire, UK).

##### Preparation of DOX‐Loaded AG

The free‐base form of DOX (3 mg) was dispersed in a cosolvent of dimethyl sulfoxide (DMSO; 10 μL) and DDW (10 μL). AG (20 mg) and DDW (80 μL) were added to the DOX solution, followed by ball milling (stainless steel bead; diameter: 5 mm; QIAGEN, Hilden, Germany) for 3 min. The mixture was diluted with DDW (1 mL) and lyophilized at –70 °C to remove DMSO. The freeze‐dried product was redispersed in DDW and filtered using a syringe filter (pore size: 0.45 μm; regenerated cellulose) to remove unloaded (precipitated) DOX. The resulting solution was then lyophilized again at –70 °C to obtain DOX‐loaded AG (DOX@AG) powder.

##### Preparation of DOX‐Loaded Nanohybrids

DOX@AG was hybridized with a lipid complex for HCC targeting. EPC (1 mg), SG (0.25 mg), and 1,2‐distearoyl‐*sn*‐glycero‐3‐phosphoethanolamine‐*N*‐[amino(polyethylene glycol)‐2000] (DSPE‐PEG‐NH_2_; 0.25 mg) were dissolved in methanol (250 μL) and dried at 65 °C under nitrogen stream to prepare the HCC‐targeting lipid complex film (SGLC). To this SGLC lipid composite, DOX@AG solution (12 mg mL^−1^) was added and homogenized via tip sonication for 1 min to produce SGLC/DOX@AG nanohybrids. A control formulation, LC/DOX@AG nanohybrids, was prepared using the same method described above, but without SG in the lipid complex film.

##### Characterization of DOX‐Loaded Nanohybrids

The mean diameter, polydispersity index, and zeta potential of the nanohybrids were measured using DLS (Zetasizer Ultra, Malvern Panalytical). To evaluate the colloidal stability of the LC/DOX@AG (F1) and SGLC/DOX@AG (F2), each formulation was diluted with 10 mM PBS (pH 7.4) to a protein concentration of 10 mg/mL and incubated in a shaking water bath (50 rpm) at 37 °C. The mean diameter change was monitored at predetermined time intervals (0, 1, 2, 4, 8, 12, and 24 h) using DLS. The morphology of the formulations was observed using TEM (JEM 2100, JEOL, Tokyo, Japan), wherein F1 and F2 were stained with 2% w/v phosphotungstic acid and applied to a copper grid covered with a thin carbon film.

##### Determination of DOX Concentration in Nanohybrids

The DOX content of DOX@AG was first quantified by high‐performance liquid chromatography (HPLC) analysis, wherein DOX was extracted from DOX@AG by diluting 50 times with DMSO. The samples were then centrifuged at 16 000 *g* for 5 min at 20 °C,^[^ ^19^, ^46^ ^]^ and the supernatant was analyzed using an HPLC system (1260 Infinity 2, Agilent Technologies, Santa Clara, CA, USA), equipped with a reverse phase C‐18 column (Kinetex, 250 × 4.6 mm, 5 μm; Phenomenex, Torrance, CA, USA). The mobile phase consisted of a potassium phosphate buffer (10 mm, pH 2.5) and acetonitrile with 0.1% triethylamine (70:30, v/v). The flow rate was set to 1.0 mL min^−1^. The injection volume was 20 μL, and the eluent was monitored at the excitation and emission wavelengths of 480 and 565 nm, respectively.^[^ ^47^, ^48^, ^49^ ^]^ DOX content and entrapment efficiency (EE) of lyophilized DOX@AG were calculated as follows:

(1) $$\text{Drug content of DOX}@\text{AG }\left(\%\right)\;=\frac{\text{amount of DOX in DOX}@\text{AG }}{\text{amount of DOX}@\text{AG }}\times 100$$

(2) $$\text{EE of DOX}@\text{AG }\left(\%\right)\;=\frac{\text{actual amount of DOX in DOX}@\text{AG }}{\text{theoretical amount of DOX in DOX}@\text{AG }}\times 100$$

The DOX concentration in nanohybrids was calculated based on the drug content of DOX@AG:

(3) DOX concentration in nanohybrids=Drug content of DOX@AG/100×DOX@AG concentration in nanohybrids

##### Cell Culture

HUVECs (Lonza, Basel, Switzerland) were cultured in endothelial growth medium (Lonza), and cells from passages 2 to 5 were used for the experiments. Normal HLFs (Lonza) were cultured in fibroblast growth medium (FGM‐2, Lonza), and cells from passages three to six were used for the experiments. HepG2 cells (KCLB88065) were cultured in the minimum essential medium (MEM, WELGENE, Gyeongsan, Republic of Korea). Hep3B (KCLB 88064) cells were cultured in the Dulbecco's modified Eagle medium (DMEM, WELGENE). Huh7 (KCLB 60104) cells were cultured in the Roswell Park Memorial Institute (RPMI 1640, WELGENE). Hepa1‐6 mouse liver cancer cell lines (ATCC CRL‐1830) were cultured in DMEM (Gibco, Thermo Fisher Scientific) supplemented with 10% fetal bovine serum (FBS; Gibco, Thermo Fisher Scientific) and 1% penicillin–streptomycin (Gibco, Thermo Fisher Scientific) at 37 °C in a humidified atmosphere with 5% CO_2_. Cells were passaged when they reached ≈80% confluency.

##### In Vitro Release Study

The DOX release profiles of F1 and F2 were investigated. Each formulation (150 μL) was loaded into a mini‐GeBAflex tube (molecular weight cutoff: 4–6 kDa; Gene Bio‐Application Ltd., Kfar Hanagide, Israel), which was then immersed in PBS (pH 5.5, 6.8, and 7.4) and agitated in a shaking incubator at 37 °C (50 rpm). Aliquots (200 μL) were collected from the media at the predetermined times (1, 2, 4, 6, 8, 24, 48, and 72 h) and fresh medium (200 μL) was replenished at each time point. The amount of DOX released was determined using HPLC as described above.

##### Cell Cytotoxicity Assay

The cytotoxicity of DOX‐loaded and blank formulations (without DOX) was evaluated using the cell counting kit‐8 (CCK‐8) assay. HepG2 cells were seed on 96‐well plates at a density of 1.0 × 10^4^ cells per well and incubated with DOX‐loaded (as DOX, 0.2, 0.5, 1, 2, 5, and 10 μg mL^−1^) or blank formulations (as formulation, 2, 5, 10, 20, 50, and 100 μg mL^−1^) for 24, 48, and 72 h at 37 °C. At the indicated time points, absorbance was measured at 480 and 600 nm (background) on a Tecan Spark microplate, and half‐maximal inhibitory concentration (IC_50_) was calculated using CompuSyn software (Version 1.0; ComboSyn, Inc., NJ, USA).

##### Cellular Uptake Study

HepG2 cells were treated 6 × 10^5^ (2 mL) in 6 well plates. The groups were also set up as DOX@BSA, DOX@AG, DOX@AG co‐treated with d‐glucosamine (75 μg mL^−1^), F1, F2, and F2 cotreated with d‐glucosamine (75 μg mL^−1^). All formulation (1 mL) were added to each well at the same DOX concentration (50 μg mL^−1^). The formulations were incubated for 4 h after the treatment. 0.5% trypsin‐EDTA (1 mL) and 2% FBS in PBS (1 mL) were used for flow cytometry. HepG2 spheroids were cultured in SpheroFilm (INCYTO, Cheonan, Republic of Korea). The cell pellet was resuspended in growth medium, and 3 × 10^6^ cells/3 mL were thawed in 300 μm SpheroFilm and incubated in cell culture media according to the manufacturer's protocol. The spheroid cell for 3–4 days and treat with DOX, F1, and F2, were diluted at 10 μm for DOX concentration in MEM media. At 24, 48 and 72 h after drug treatment, the spheroids were washed by PBS solution 3 times and incubated in Calcelin‐AM and Hoechst 33342 solution for 10 min. Doxorubicin, Calcein‐AM, and Hoechst were measured using a confocal microscope (LSM700, Zeiss, Jena, Germany) at the same Ex and Em at each time point.

##### Animals and Husbandry

Male C57BL/6 mice (6–7 weeks old, weighing 18–20 g) were purchased from Orient Bio (Sungnam, Republic of Korea) and used after a 12 day acclimatization period. The animals were housed five per polycarbonate cage under controlled temperature (20–25 °C) and humidity (40–45%) with a 12:12 h light/dark cycle, fed a normal rodent pellet diet, and supplied with water ad libitum. All animals were treated in accordance with the Guidelines for the Care and Use of Laboratory Animals issued by Chungnam National University (IRB No.: CNU‐01051).

##### In Vivo Biodistribution in Xenograft Model

Near‐infrared fluorescence (NIRF) imaging was performed in HepG2 xenograft mice using Cy5.5‐tagged nanohybrids. Cy5.5‐NHS (100 μg) and AG (100 mg) were incubated in DDW for 12 h and dialyzed against DDW (pH 8.6, adjusted with NaOH) for 24 h, followed by lyophilization at –70 °C to produce Cy5.5‐tagged AG. This Cy5.5‐tagged AG was used as the blank sample in imaging experiments, representing the protein formulation without lipid coating. Cy5.5‐tagged F1 and F2 were prepared by the same nanohybrid preparation method above, except for the use of Cy5.5‐tagged AG. HepG2 cells (5.0 × 10^6^ cells) were suspended in OptiMEM and subcutaneously injected into the upper right back region of mice. The tumor volume (V, mm^3^) was calculated with the following formulation: *V* = 0.5 × longest diameter × (shortest diameter)^2^. After the tumor volume reached ≈100 mm^3^, Cy5.5‐tagged formulations were injected into xenograft mice intravenously the whole‐body NIRF was monitored under isoflurane anesthesia at 1, 2, 6, and 24 h postinjection using a VIQUE in vivo smart imaging system (Vieworks, Anyang, Republic of Korea) equipped with CleVue software (Vieworks). For ex vivo analysis, the mice were sacrificed 24 h after injection, and the liver, kidney, spleen, lung, heart, and tumors were sampled and analyzed using the same imaging instrument.

##### In Vivo Antitumor Efficacy in Xenograft Model

The antitumor efficacy of DOX‐loaded nanohybrids was evaluated in the HepG2 xenograft model described above. The mice were randomly divided into four groups: control (no intervention), DOX solution, F1, and F2, and intravenously administered each intervention at a DOX dose of 5 mg kg^−1^ on days 1, 3, 5, 7, 9, and 11. The tumor volume and body weight were monitored daily. Tumor tissues were dissected and weighed on day 12.

##### Preparation of Orthotopic Liver Cancer Model

C57BL/6 mice, 6 weeks old, were purchased from Orient Bio and acclimated for 1 week prior to experimentation. To establish an orthotopic liver cancer model, Hepa1‐6 cells were harvested and resuspended in serum‐free DMEM at a concentration of 1 × 10^7^ cells mL^−1^. The cell suspension was then mixed with an equal volume of Matrigel (Corning, Corning, NY, USA) to achieve a final concentration of 1 × 10^6^ cells/50 μL. Matrigel–cell mixture was kept on ice to prevent premature gelation. Mice were anesthetized with isoflurane inhalation (2–3% for induction, 1–2% for maintenance, in 100% oxygen). The abdomen was sterilized with 70% ethanol, and a small midline incision was made to expose the liver. A 50 μL aliquot of the Hepa1‐6/Matrigel mixture (containing 1 × 10^6^ cells) was injected directly into the left lobe of the liver using a 29 gauge needle. The injection was performed slowly to prevent leakage. After injection, the needle was kept in place for an additional 10 s to allow the mixture to solidify and prevent backflow. The abdominal incision was closed with 6‐0 polypropylene sutures (Ethicon, Johnson & Johnson). Mice were allowed to recover in a warmed environment and were monitored until fully awake.

##### Blood Biochemistry

Blood samples were collected from the SGLC‐AG NP‐injected HepG2 xenograft model, and serum was separated by centrifugation at 16 000 *g* for 10 min. Blood biochemistry data were analyzed using a Fuji DRI‐CHEM NX500i (Fujifilm Corp., Tokyo, Japan) at the Chiral Material Core Facility Center at Sungkyunkwan University. Serum alanine transaminase (ALT), aspartate aminotransferase (AST), lactate dehydrogenase (LDH), blood urea nitrogen (BUN), and creatinine (SCr) levels were determined.

##### Microfluidic Chip Fabrication

The microfluidic device was fabricated using metal frames and microneedles as templates. A10:1 (w/w) mixture of polydimethylsiloxane (PDMS) (Sylgard 184, Dow Corning, Midland, MI, USA) was poured onto the metal frame with the needle inserted and degassed under vacuum. The assembly of the PDMS‐filled metal frame and glass was cured for 1 h in an oven at 80 °C. After separation of the frame and glass substrate, the microneedles were removed from the cured PDMS. Part of the hydrogel for injection was cut with a 4 × 5 mm square punch, and subsequently, a flat PDMS sheet was bonded to the punched PDMS after oxygen plasma treatment. The reservoirs for the cell culture medium and the holes for hydrogel injection were punched using 8 and 1 mm biopsy punches (Kai Medical, Tokyo, Japan), respectively. After inserting the needle into the PDMS chip, the dust was removed using a residue‐free tape, and the PDMS chip was assembled with a cover glass (50 × 70 mm, Matsunami Glass IND LTD, Osaka, Japan) after treatment with oxygen plasma (FEMTO Science, Hwaseong, Republic of Korea).

##### Engineered Blood Vessel Experiment

Engineered blood vessels were fabricated and permeability assays were performed as described previously.^[^ ^50^ ^]^ The permeability assay was performed by introducing 40 kDa FITC‐Dextran solution (10 μm, Sigma–Aldrich) into the perfusable blood vessel and monitoring the molecular transport using a confocal microscope (LSM700, Zeiss). The acquired images were color‐mapped using custom‐written MATLAB codes (MathWorks, CA, USA) and the fluorescence intensity was displayed in an arbitrary unit. The mean fluorescence intensity values across the microchannels were analyzed using custom‐written MATLAB (MathWorks) codes.

##### Western Blotting

Whole‐cell extracts from HepG2 cells were lysed using RIPA buffer containing protease inhibitors (0.17 mg mL^−1^ PMSF, 2 μg mL^−1^ leupeptin, and 0.7 μg mL^−1^ aprotinin), followed by SDS–PAGE analysis. The following primary antibodies were used: anti‐caspase‐3 (Cat. #9662S, CST), anti‐caspase‐8 (Cat. #ab25901, Abcam), anti‐caspase‐9 (Cat. #ab202068, Abcam), anti‐AKT (Cat. #4691S, CST), anti‐phospho‐AKT (Ser473) (Cat. #9271T, CST), anti‐phospho‐AKT (Thr308) (Cat. #92757, CST), anti‐LDH‐B (Cat. #sc‐100775, Santa Cruz), anti‐VE‐cadherin (Cat. #2158S, CST), anti‐ICAM‐1 (Cat. #62133, CST), anti‐F‐actin (Cat. #A22287, Abcam), and DAPI (Cat. #62247, Thermo Scientific). Immunodetection was performed using an enhanced chemiluminescence system, and bands were analyzed with a WSE‐6100 LuminoGraph I (ATTO, Japan).

##### WST‐1 Cell Proliferation Assay

WST‐1 reagent (10 μL/well) was added in 96‐well plates and incubated at 37 °C with 5% CO_2_ for 2 h. At the indicated time points, the absorbance was measured at 480 and 600 nm (background) using a Tecan Spark microplate reader (Männedorf, Switzerland).

##### Statistical Analysis

All the in vitro and in vivo data were analyzed via two‐tailed unpaired *t*‐test using the GraphPad Prism 8 software, the prepared sample sizes were *n* ≥ 3, and the statistical significance was set at *p* < 0.05. Detailed information on each experiment is provided in the figure legends. All data normalization processes were performed according to the manufacturer's instructions. Data transformation and outlier evaluation were not performed.

## Conflict of Interest

The authors declare no conflict of interest.

## Author Contributions

**Hyelim Kim**: writing—original draft; project administration; methodology; investigation; formal analysis; data curation; conceptualization. **Han Sol Lee**: writing—original draft; project administration; methodology; investigation; formal analysis; data curation; conceptualization. **Tae‐Wan Kwon**: methodology; data curation; conceptualization. **Donghyuk Seo**: methodology; formal analysis. **So‐Yeol Yoo**: methodology; formal analysis; formal analysis; data curation. **Sang Kyum Kim**: methodology; formal analysis. **Hee Ho Park**: methodology; formal analysis. **Seung‐Woo Cho**: writing—review and editing. **June Hong Ahn**: writing—review and editing. **Wonhwa Lee**: writing—review and editing. **Jae‐Young Lee**: writing—review and editing; supervision; project administration; funding acquisition; conceptualization. **Hong Nam Kim**: writing—review and editing; supervision; project administration; funding acquisition; conceptualization. **Hyelim Kim** and **Han Sol Lee** contributed equally to this work.

## Supporting information

Supplementary Material

## Data Availability

The data that support the findings of this study are available on request from the corresponding author. The data are not publicly available due to privacy or ethical restrictions.
