# Supplementary material for: Targeted Nanocomplex Delivery for Protecting Vascular Integrity and Enhancing Anticancer Effects in Hepatocellular Carcinoma
Source: Small Sci. 2025 Jul 25;6(1):e202400616. doi: 10.1002/smsc.202400616 (PMC12854391; doi:10.1002/smsc.202400616)
Supplement: Supplementary file 1 — Supplementary Material [file SMSC-6-e202400616-s001.pdf]

Supporting Information

Targeted Nanocomplex Delivery for Protecting Vascular Integrity and Enhancing Anti-Cancer Effects in Hepatocellular Carcinoma

Hyelim Kim[a,b,1], *Han Sol Lee*[c,1], *June Hong Ahn*[d], *Tae-Wan Kwon*[e], *So-Yeol Yoo*[e,f], *Donghyuk Seo*[g], *Sang Kyum Kim*[e], *Hee Ho Park*[h], *Seung-Woo Cho*[b,*] *Wonhwa Lee*[g,*], *Jae-Young Lee*[e,f,*], *Hong Nam Kim*[a,i,j,k,*]

Hyelim Kim, Hong Nam Kim

Brain Science Institute, Korea Institute of Science and Technology (KIST), Seoul, 02792, Republic of Korea

Email: hongnam.kim@kist.re.kr (H.N.K.)

Hyelim Kim, Seung-Woo Cho

Department of Biotechnology, Yonsei University, Seoul, 03722 Republic of Korea

Email: seungwoocho@yonsei.ac.kr

Han Sol Lee

College of Pharmacy, Chosun University, Gwangju 61452, Republic of Korea

June Hong Ahn

Division of Pulmonology and Allergy, Department of Internal Medicine, College of Medicine, Yeungnam University and Regional Center for Respiratory Diseases, Yeungnam University Medical Center, Daegu 42415, Republic of Korea

Tae-Wan Kwon, So-Yeol Yoo, Sang Kyum Kim

College of Pharmacy, Chungnam National University, Daejeon 34134, Republic of Korea

So-Yeol Yoo, Jae-Young Lee

College of Pharmacy and Research Institute of Pharmaceutical Sciences, Seoul National University, Seoul 08826, Republic of Korea

Email: lee.jy@snu.ac.kr

Donghyuk Seo, Wonhwa Lee

Department of Chemistry, Sungkyunkwan University, Suwon, Gyeonggi-do 16419, Republic of Korea

Email: wonhwalee@skku.edu

Hee Ho Park

Department of Biotechnology, College of Life Sciences and Biotechnology, Korea University, 145, 02841 Republic of Korea

Hong Nam Kim

School of Mechanical Engineering, Yonsei University, Seoul 03722, Republic of Korea

Yonsei-KIST Convergence Research Institute, Yonsei University, Seoul 03722, Republic of Korea

Division of Bio-Medical Science & Technology, KIST School, University of Science and Technology, Seoul 02792, Republic of Korea

[1]These authors contributed equally to this work.

Corresponding authors.

Email: seungwoocho@yonsei.ac.kr (S. -W. J.), wonhwalee@skku.edu (W.L.), lee.jy@snu.ac.kr (J.-Y. L.), hongnam.kim@kist.re.kr (H.N.K.)

1. Supplementary tables

Table S1. Composition of lipid hybridized fomulations.

Composition	LC/DOX@AG (F1)	SGLC/DOX@AG (F2)
Lipoid E100 (EPC)	1 mg	1 mg
DSPE-PEG-NH_2	0.25 mg	0.25 mg
Stearyl glycyrrhetinate (SG)	-	0.25 mg
DOX@AG	12 mg	12 mg

Groups	IC_{50}*		
	24 h	48 h	72 h
DOX solution	6.66 ± 1.38	1.98 ± 0.67	0.96 ± 0.15
F1	1.54 ± 0.04	1.17 ± 0.07	1.12 ± 0.08
F2	1.51 ± 0.12	0.94 ± 0.15	0.70 ± 0.10

Table S2. IC_{50} values of DOX solution and formulations. Data are presented as mean ± SD (n = 6).

*The unit of IC_{50} values is μg/mL.

Table S3. Blood biochemistry parameters after anti-cancer test to mice

Parameters (reference ranges)	Control	DOX	F1	F2
ALB (3.4–3.9 g/dL)	2.70 ± 0.14	2.95 ± 0.21	2.50 ± 0.00	2.50 ± 0.26
AST (53–131 U/L)	321.50 ± 64.35	314.50 ± 62.96	474.00 ± 109.14	215.33 ± 59.23
ALT (22–61 U/L)	62.00 ± 4.24	52.00 ± 8.49	51.00 ± 3.61	60.67 ± 15.31
SCr (0.0–0.2 mg/dL)	0.21 ± 0.12	0.21 ± 0.05	0.20 ± 0.02	0.28 ± 0.04
BUN (12.2–17.5 mg/dL)	23.00 ± 3.39	21.50 ± 0.71	23.77 ± 1.00	28.33 ± 4.38
LDH (U/L)	900 ± 0.00	900 ± 0.00	900 ± 0.00	551.67 ± 34.20

ALB: albumin, AST: aspartate aminotransferase; ALT: alanine transaminase; SCr: serum creatinine, BUN: blood urea nitrogen, LDH: lactate dehydrogenase.

2. Supplementary figures

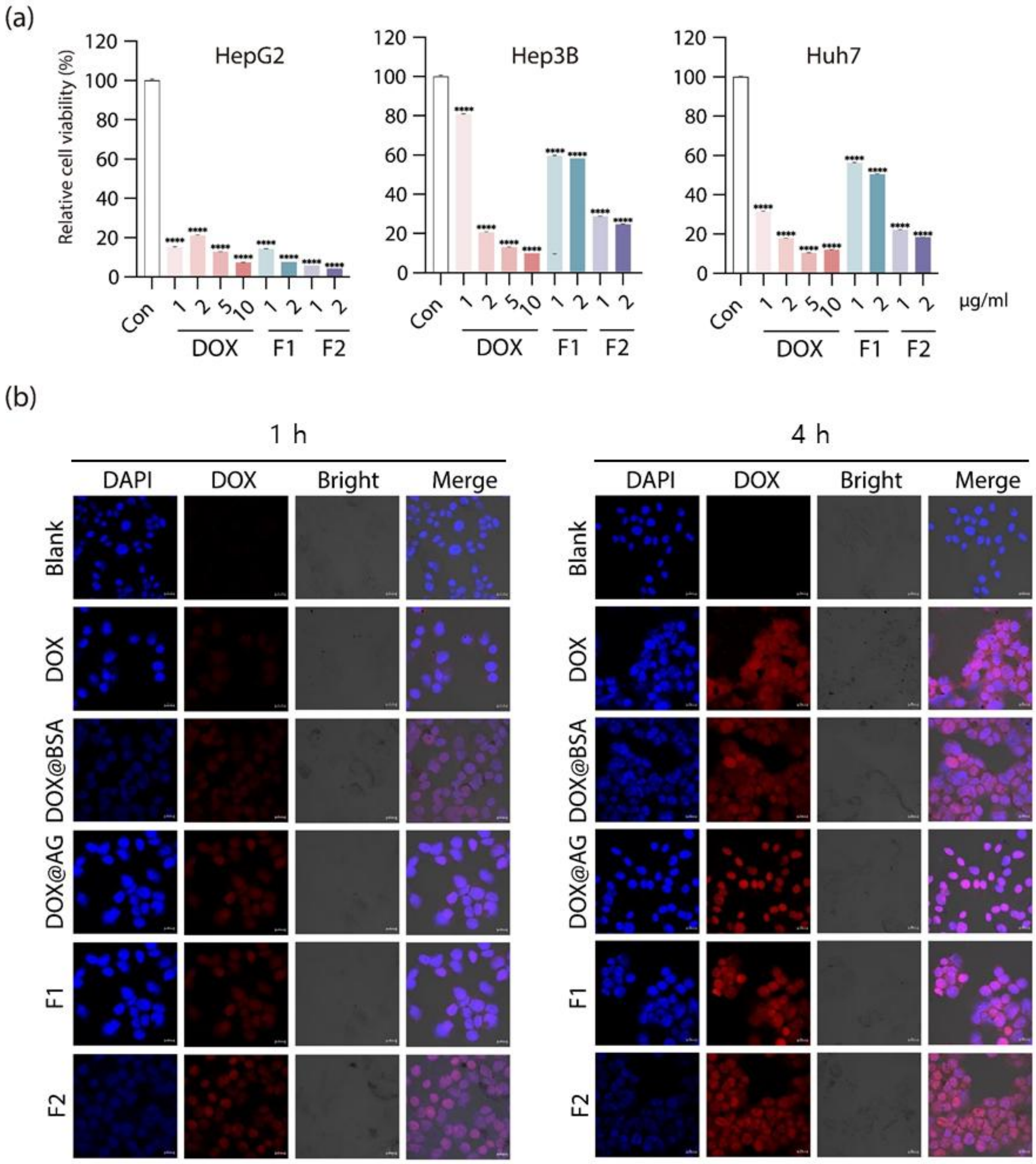

Figure S1. Anti-cancer effects and cellular uptake of F1 and F2 on hepatocellular carcinoma cell lines. (a) HepG2, Hep3B, and Huh7 were cultured in 96-well plates with a cell density of 1 x 10^4 cells/well. DOX, F1, and F2 were treated for 24 hours, and cell viability was evaluated by MTT assay. Data are presented as mean ± SEM. *P*-values were calculated using a *t*-test. **p* < 0.05, ***p* < 0.01, ****p* < 0.001, and *****p* < 0.0001. (b) Confocal images of HepG2 treated with free DOX, DOX@BSA, DOX@AG, F1, and F2. DAPI (blue) and DOX (red) visualize nuclei and drug localization, respectively. Scale bar = 20 µm.

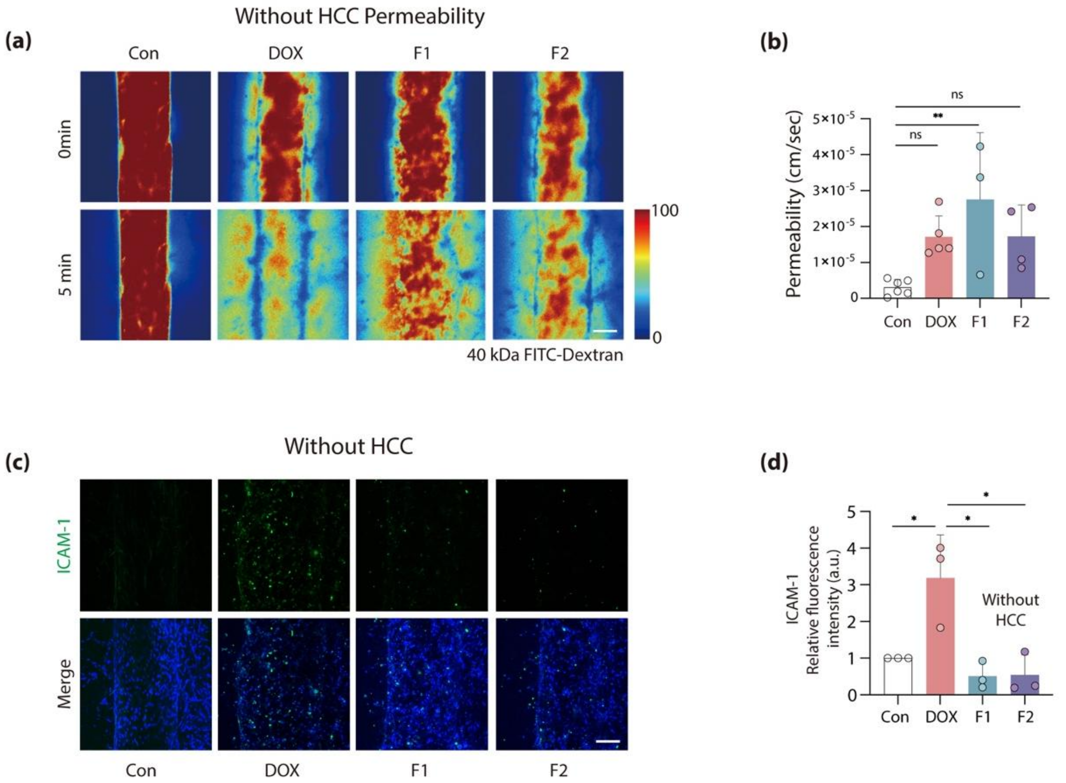

Figure S2. Evaluation of vascular disruption and inflammation in vascularized cancer on a chip. (a) Transendothelial permeability of microvasculature in the absence of HCC spheroid. Leakage of 40 kDa FITC-dextran from the microvasculature was visualized. (b) Quantified transendothelial permeability from. (c) Immunofluorescence imaging of vascular inflammation marker. ICAM-1 (green), nuclei (blue) Scale bar: 100 μm. (d) Quantitative fluorescence intensity of ICAM-1. ($n \geq 4$ for each group) The experiment was performed at least three times with replicates. Data are presented as mean ± standard error of the mean (SEM). P-values are calculated using an ANOVA (A-H). *$p < 0.05$, **$p < 0.01$. ***$p < 0.01$. ****$p < 0.01$. Scale bar, 100 μm. (significance is indicated by * for $p < 0.05$; P-values are calculated using an ANOVA).

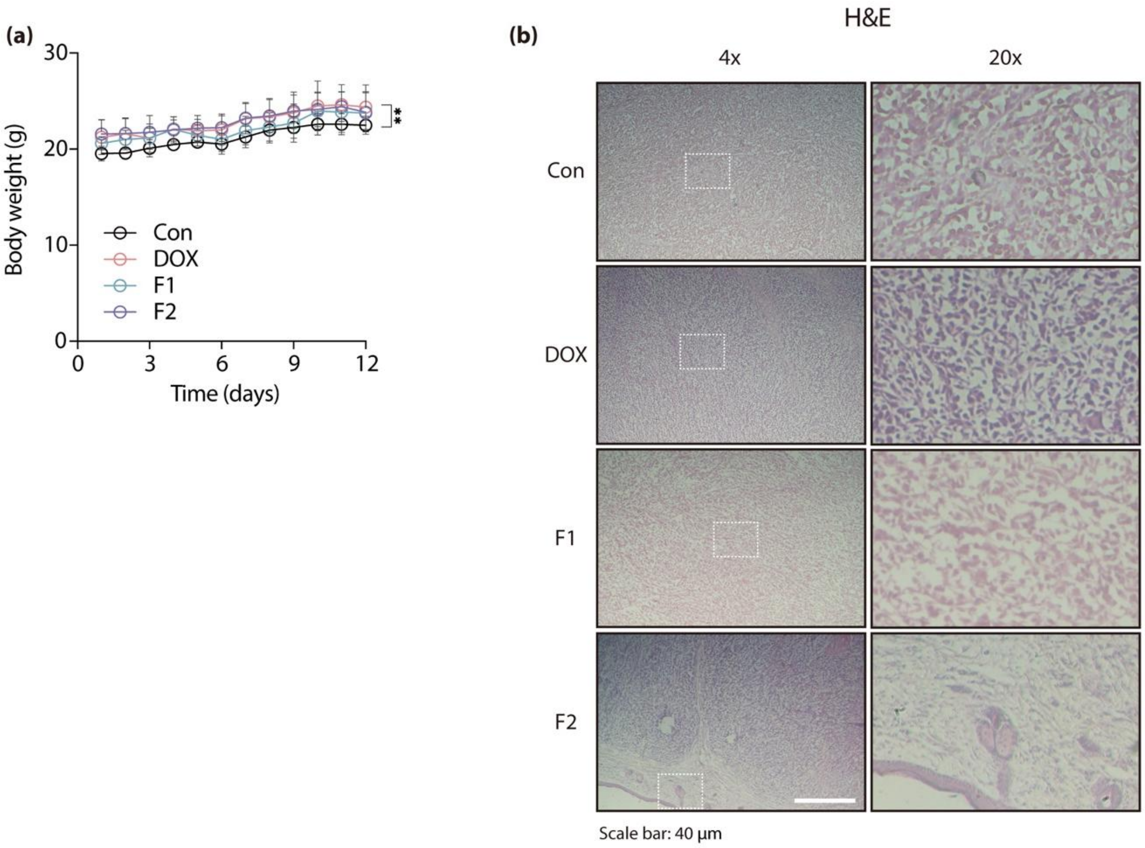

Figure S3. Validation of anti-cancer effect in xenografted mouse model. (a) Body weight of the tumor bearing mouse. (n = 5 for each group) (b) The hematoxylin & eosin staining of xenograft mice tumor tissue. Statistical significance is indicated as $^{***}p < 0.001$. Scale bar: 40 μm.
